# To see or not to see: the parallel processing of self-relevance and facial expressions

**DOI:** 10.1186/s41235-023-00524-8

**Published:** 2023-11-22

**Authors:** Tuo Liu, Jie Sui, Andrea Hildebrandt

**Affiliations:** 1 Division for Psychological Methods and Statistics, Department of Psychology, Carl von Ossietzky Universität Oldenburg, Oldenburg, Germany; 2 School of Psychology, King’s College, University of Aberdeen, Aberdeen, Scotland, UK

**Keywords:** Self-relevance, Facial emotion, Self-concept

## Abstract

The self, like the concept of central "gravity", facilitates the processing of information that is directly relevant to the self. This phenomenon is known as the self-prioritization effect. However, it remains unclear whether the self-prioritization effect extends to the processing of emotional facial expressions. To fill this gap, we used a self-association paradigm to investigate the impact of self-relevance on the recognition of emotional facial expressions while controlling for confounding factors such as familiarity and overlearning. Using a large and diverse sample, we replicated the effect of self-relevance on face processing but found no evidence for a modulation of self-relevance on facial emotion recognition. We propose two potential theoretical explanations to account for these findings and emphasize that further research with different experimental designs and a multitasks measurement approach is needed to understand this mechanism fully. Overall, our study contributes to the literature on the parallel cognitive processing of self-relevance and facial emotion recognition, with implications for both social and cognitive psychology.

## Introduction

As highly social beings, humans have to deal with huge amounts of information in their social interactions, both from themselves and from others. One of the earliest robust findings in cognitive psychology indicates that self-related information is preferentially processed over other kinds of information (Rogers et al., 1977). For instance, it is easier to recognize one's own name (Bargh, 1982), self-voices (Candini et al., 2014) and self-body parts (Frassinetti et al., 2011) compared to those of others. However, while there is broad evidence suggesting biased processing of self-related information, literature pointed at methodological weaknesses in research on the self in general, which suggests that the effect may be driven by the effect of learning self-related information over a long period of time, such as one's name and face (Sui & Gu, 2017). Thus, a troublesome familiarity confound underlies the interpretation of the self-prioritization effect. As a result, it is impossible to disentangle the cause of self-related information from the effect of familiarity and overlearning. Over the last decade, numerous researchers have found that this bias towards self over others occurs not only for consolidated information over the long term, but also for information that is temporarily associated with the self, even within the last few minutes (Sui et al., 2012). Using a learning approach to associate the self with unfamiliar novel things temporarily, researchers have elegantly ruled out the confounding influence of familiarity and overlearning (Lee et al., 2021; Sui et al., 2015).

To distinguish this prioritized processing from self-related information in general, here we use the term “self-relevance” to indicate the benefit of processing information that is temporarily related to the self. A study by Sui et al. (2012) provided the first direct empirical evidence for this new approach, named the self-association paradigm. Participants were asked to associate social labels (e.g., self or other) with neutral geometric shapes in the instruction. A subsequent perception-matching task indicated that shapes associated with self-labels were judged faster and more accurately than those associated with other labels, even if this association was wholly temporal. This approach has been conceptually replicated and combined with different specific tasks across different cognitive domains, such as attention (Dalmaso et al., 2019), decision-making (Sui et al., 2016), and action control (Desebrock et al., 2018; Frings & Wentura, 2014). This growing body of evidence reflects the high degree of the malleability of self-relevance.

Facial expressions of emotion belong to the most crucial cues in social interactions (Van Kleef, 2009). In fact, research has shown that more than 50% of emotional information is transmitted through facial expressions during social communication (Lapakko, 1997). From a developmental perspective on social cognition, it is widely accepted that the processing of self-related information is related to the processing of emotional facial expressions (Happé et al., 2017). In addition, evidence from various sub-disciplines, including evolutionary psychology (Conway et al., 2019; Gonzalez-Liencres et al., 2013), clinical psychology (Uddin, 2011; Williams, 2010), social psychology (Ma & Han, 2010), and cognitive neuroscience (Northoff, 2016; Scheller & Sui, 2022), suggests a potential relationship between the self and the processing of emotional facial expressions. Taken together, these studies suggest that the self plays a vital role in the socio-cognitive processing of emotional facial expressions.

Despite these effects of self-relevance on cognition (Sui & Humphreys, 2017), the potential impact of self-relevance on emotional expression has received relatively little attention. A convenient forward citation search of the paper by Sui et al. (2012) using Google Scholar yielded only six publications out of 316 citations related to emotional facial expressions. Most of these six studies provide only indirect evidence of how self-relevance affects the processing of emotional facial expressions. For instance, in Constable et al. (2021) and McIvor et al. (2021), participants associated social labels with happy or sad expressions and performed a perceptual matching task with the label-drawing pairs thereafter. Although more accurate recognition of self-associated facial expressions was found as compared to those associated with other labels, these findings could not directly support the claim that self-relevance improves facial expression processing. This is because the perceptual matching task measures the magnitude of self-association only, rather than the cognitive performance to process emotional expressions (Woźniak & Knoblich, 2019). Despite the fact that these studies provide some insight into the relationship between self- and facial emotion-related processing, further direct evidence is needed by using a specific task that measures facial expression recognition.

Recent studies (Payne et al., 2017; Woźniak & Hohwy, 2020; Woźniak & Knoblich, 2019; Woźniak et al., 2018) have replicated the biased processing in the case of self-relevant facial stimuli. These studies extended the evidence on prioritized self-associative processing to the domain of facial stimuli, showing that self-association with an unfamiliar face can improve performance on a perceptual matching task of the same faces. Furthermore, by means of event-related potentials (ERP), Woźniak et al. (2018) found that the perception of self-associated previously unfamiliar faces led to the same modulation of facial processing-related ERPs as the perception of one's own face. This result was interpreted not only as evidence for the formation of self-relevance with these faces but also as a support for the idea that the self-relevance could directly enhance facial processing, which is an essential stepping-stone for the development of facial emotion recognition (Happé et al., 2017). Given that face processing and facial emotion recognition are highly correlated abilities (Hildebrandt et al., 2015), exploring the influence of self-relevance on facial emotion recognition using the self-association paradigm is particularly meaningful. In addition, some of the previous studies have been criticized given their low power and convenience samples. For example, the largest sample size in the above-mentioned studies was 31, and nearly all participants were students. To remedy this problem it has been recommended to collect larger samples of participants with diverse backgrounds (Camerer et al., 2018). As a first goal, we thus aimed to replicate the effect of self-association using facial stimuli in a larger and more diverse sample.

Usually, six basic emotions are studied in facial emotion recognition research: Happiness, Surprise, Fear, Sadness, Disgust, and Anger. Cross-cultural research has shown that these prototypical expressions can be accurately identified and distinguished from each other (Elfenbein & Ambady, 2002). However, several studies mentioned above have investigated the relationship between self-relevance and facial emotion processing only on some of these basic emotion categories. For example, Cunningham et al. (2022) only examined faces expressing anger. Some studies used more than one emotion, mainly happiness and sadness expressions (Feldborg et al., 2021; McIvor et al., 2021; Stolte et al., 2017a; Yankouskaya & Sui, 2021). Indeed, happiness and sadness are the two expressions at the opposite ends of the positive–negative valence spectrum (Bimler & Kirkland, 2001). However, there is evidence of a more fine-graded emotion category-related specificity in emotion recognition (Kirita & Endo, 1995; Kirouac & Doré, 1983; Wells et al., 2016). Studies showed different accuracy and speed levels when processing different facial expressions of emotion. For example, there is a large literature suggesting that happy faces are more accurately recognized than other facial expressions (e.g., Kirita & Endo, 1995; Stolte et al., 2017b; Svard et al., 2012), while fear expressions are difficult to recognize and are often confused with sadness, given the overlapping facial action units between these expressions (Guarnera et al., 2015). Thus, previous studies' generalizability may be limited by their narrow focus on a few emotion categories. Methodological studies long recommended using all basic emotion categories when measuring facial emotion recognition ability (O’Sullivan & Ekman, 2004). Therefore, for a more complete picture, we here aim to investigate whether self-association influences the processing of facial expressions of emotion across all basic emotion categories.

Accordingly, the aim of our study was twofold. First, we attempted to replicate the experiment of self-relevance on facial processing using a large and diverse sample. To achieve this goal, we recruited participants with diverse demographic backgrounds over an online crowd-working platform. Previous validation studies have demonstrated that the data quality obtained from online crowd-working platforms is comparable to (Armitage & Eerola, 2020), or even better than (Hauser & Schwarz, 2016) those collected in a lab. We used a perceptual matching task to examine the effect of self-relevance using facial stimuli and followed the procedure used in previous studies (see details below). Given replication success, we expect that after the association learning, participants will have a more accurate and faster response to the faces associated with the self, as compared to those with other labels.

Given the role of self-related information processing and emotional facial expression processing in social communication (Bayer et al., 2017; Lee et al., 2023), we aim to investigate the potential influence of self-relevance on the recognition of emotional expressions. Specifically, we investigate whether the effect of self-relevance extends beyond mere facial processing to influence subsequent facial emotion recognition. To comprehensively investigate this association, we used a facial expression recognition paradigm with emotional composite faces of all six basic emotions (see below). This paradigm has been repeatedly used as a measure of emotion expression recognition performance (Calder et al., 2000; Durand et al., 2007; Hildebrandt et al., 2015; McKendrick et al., 2016; Meaux & Vuilleumier, 2016; Tanaka et al., 2012; Wilhelm et al., 2014). We hypothesized a more accurate and faster response towards the emotional expressions displayed by faces associated with a self-label as compared to those with other labels. We further expected a difference between emotion categories in line with the above-elaborated category specificity in emotion recognition ability. Finally, we anticipate an interaction between self-relevance and emotional categories.

## Method

### Participants

The data reported in this study were collected from 302 adult participants enrolled in a larger study investigating socio-emotional abilities and self-concept. All participants were recruited via the Prolific platform (www.prolific.co) in August 2021. To be eligible for participation, individuals were required to be currently residing in the UK, possess a near-native level of English knowledge, and report normal or corrected-to-normal vision. Three participants were excluded due to incomplete responses. Therefore, the final sample consisted of *N* = 299 participants, with 44% identifying as female, 54% as male, and 2% identifying as non-binary. The mean age of the sample was 32.14 years (SD = 11.29, range from 18 to 75), and the participants had a reasonably heterogeneous educational background: 26.76% held a high school degree, 55.18% held an associate or bachelor's degree, and 18.06% held a degree higher than a bachelor's degree. The study was reviewed and approved by the Committee of Ethics of the [Double Blind for the review process]. All participants provided informed consent and received a monetary compensation of 8.5 pounds for their participation.

### Stimulus material

All face photographs were taken from a study conducted by Wilhelm and colleagues (2014) and consisted of eight models (four biological females and four biological males). Additionally, the photograph of an additional model was used to create stimuli for the practice trials. None of the models had any distinctive features, such as makeup, piercings, or glasses, and all models were photographed under identical lighting and background conditions for consistency. To ensure the emotional salience of the stimuli, all photos of emotional expressions were evaluated and selected by trained researchers, additionally using the FaceReader software, as detailed in Wilhelm et al. (2014). Each photo was then uniformly cropped by fitting it into a vertical ellipse of 300 by 200 pixels to eliminate non-facial cues such as clothing and hair.

### Procedure

**Fig. 1:**
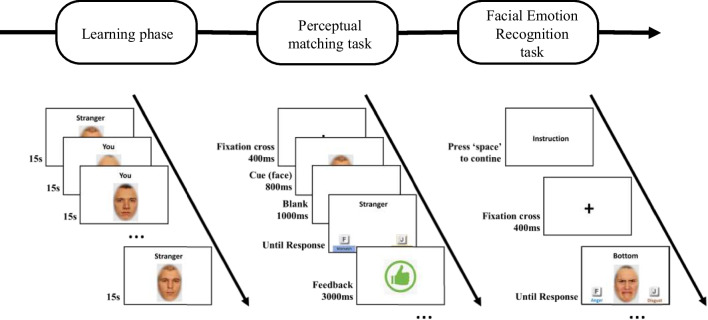
Procedure of the whole experiment

The experiment was created and hosted using the Gorilla Experiment Builder (Anwyl-Irvine et al., 2020), and participants completed the study using their own laptops or desktop computers. It consisted of three parts. In the first one, participants underwent a learning phase to memorize the associations between the neutral unknown faces and the self vs. other labels. Following this, a perceptual matching task was administered, similar to those used in previous studies investigating self-association using facial stimuli. In the third part, participants were asked to complete a specific task to measure their facial emotion recognition performance, namely the recognition task with emotional composite faces. The procedure is illustrated in detail in Fig. 1. The entire experiment lasted approximately 2 h. After completing the tasks mentioned above, participants were additionally asked to complete self-report measures of personality, as well as several ability measures of social cognition, which are beyond the scope of this study.

#### Learning phase

During the learning phase, participants were asked to associate unknown neutral faces with social labels ("You" or "Stranger"). All faces, with the associated social labels written below them, were presented on the screen one by one in random order. Participants were given 15 s to learn each face-label pairing, a timing chosen to match the one used in previous studies on self-association. In contrast to previous studies that only used one face for each label, we applied four facial models (two males and two females) counterbalanced with each social label. Therefore, participants were asked to associate themselves with four different facial identities, while associating the social label "stranger" with another set of four different facial identities. We did so in order to reduce potential confounding from a specific facial model. Each face-label pairing was repeated twice to reduce the potential memory load associated with the learning task of eight different models. Detailed instructions were shown before the beginning of the learning phase, using a practice face to ensure that participants understood the procedure.

#### Perceptual matching task

In line with previous studies, in this task participants were required to judge whether a label and a facial model displayed in a sequence matched according to what they had learned during the learning phase, or whether the label and the facial identity did not match. To ensure that participants learned the identity of the facial models and not just other features of the photographs, we used not only the photographs that were presented during the learning phase but also new photographs of the same face models with neutral expressions. These new photographs were cropped according to the same procedure as the original photographs. The only difference between the new and original photographs was a slight change in the light and visual angle (smaller than 1 degree). Each of the eight matching pairings was presented four times (two using the original photographs and two using the new photographs). Each of the eight mismatching pairs was presented four times as well. In total, this task thus consisted of 64 trials. Prior to the task, a practice trial was administered to ensure participants understood the procedure.

Each trial began with a fixation cross for 400 ms, then a face image presentation for 800 ms, followed by a delay period of 1 s. After the delay period, one of the labels ("You" or "Stranger") was displayed until participants responded using two potential response keys on the keyboard ("f" and "j"). We used the pronoun "You" here because previous studies have used this word also and showed that there was no significant difference in the pattern of results when using the pronoun "Me" or "You" (Woźniak & Hohwy, 2020). After pressing a key, visual feedback for the response (correct or incorrect) was presented, lasting 3 s. Participants were instructed to respond as quickly and accurately as possible, and the maximum response time was 5 s. If participants responded more slowly than 3 s, they received feedback to encourage quicker responses in the next trial.

#### Facial emotion recognition task with composite faces

As described in the introduction, we used a facial expression recognition paradigm with emotional composite faces to measure the emotion expression recognition performance. In this task, participants had to identify the emotion in an emotional composite face presented on one of the face halves (top vs. bottom) while ignoring the other half, which served to induce interference and increase task difficulty. These emotional composite faces were created by aligning the top and bottom halves of faces with different expressions, taken from the same person.

**Fig. 2:**
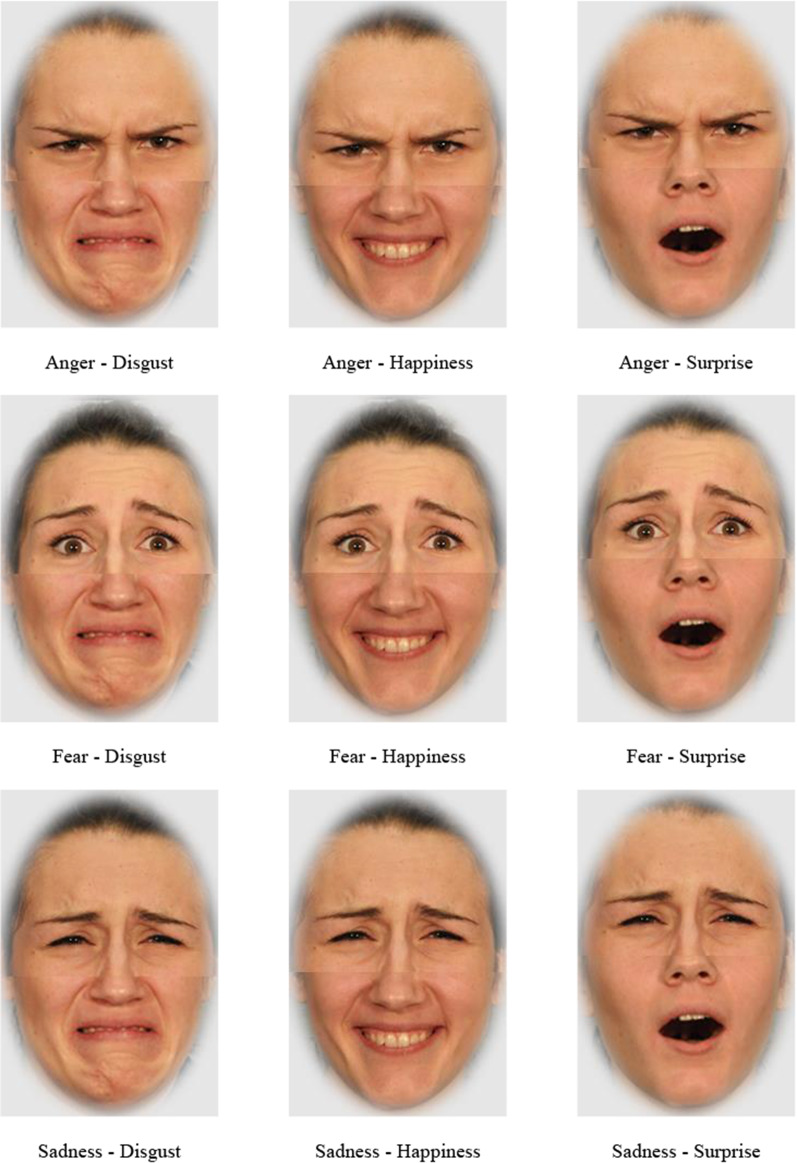
Stimuli examples used in the facial emotion recognition task

In line with previous studies, to avoid ceiling effects due to the unequal distribution of discriminative information between the upper and lower parts of the face for certain emotions, fear, sadness, and anger were only used in the upper part, while disgust, happiness, and surprise were only used in the lower part (Durand et al., 2007; Hildebrandt et al., 2015; Wilhelm et al., 2014). This resulted in nine possible composites of each model being used in the experiment. Examples of composite faces are provided in Fig. 2.

The task was a 2AFC (two-alternative forced choice) task, where participants had to press one of two keys on the keyboard (“f” and “j”) to indicate the emotion of the upper or lower halves, respectively. The targeted halves were indicated using the label “Top” or “Bottom”, displayed simultaneously on the screen with the emotional composite face. Each trial began with a fixation cross in the center of the screen for 1 s, followed by the emotional composite face and the target word, which remained on the screen until the participant responded. If participants did not respond within six seconds after stimulus onset, they were encouraged to respond more quickly in the next trial.

To counterbalance the two target words (“Top” and “Bottom”) and the nine different emotional composites, each composite face was presented twice, once for the upper half and once for the lower half. This resulted in a total of 144 trials, which were presented in two randomized blocks of 72 trials each, in random order. Participants were allowed to take a break between the blocks. To ensure that they understood the task, each block started with a practice block consisting of nine practice trials, using a practice facial model with feedback provided.

### Data analyses

The data analysis was conducted using R version 3.5.1. The code for the data analysis can be found on the website for this project at [Double Blind for the review process].

Our analysis targeted accuracy and response times (RTs) for both the perceptual matching task and the emotional expression recognition task with composite faces. To ensure that only participants who were attentive during the learning of self-association were included, we analyzed only those who responded correctly on at least 60% of the trials, which was significantly better than random guessing. We removed trials with RTs shorter than 100 ms assuming implausible cognitive processing in such a short time. Following the recommendation of Berger and Kiefer (2021), we applied an exclusion method based on *z*-scores of RTs to remove within-person outliers for each task separately. This procedure resulted in the exclusion of 1.82% of trials for the perceptual matching task and 5.28% of trials for the emotional expression recognition task, which are considered acceptable in comparison to previous studies in this domain.

#### Statistical analysis in the frequentist framework

Linear mixed models (LMMs) and generalized linear mixed models (GLMMs) were applied separately to predict response accuracy and RTs in each task. The LMMs were fitted using the lmer function from the lmerTest 3.1.3 package, while the GLMMs were fitted using the glmmTMB function from the glmmTMB 1.1.3 package. The RTs of correct trials were modelled using LMMs with a log transformation, although untransformed RTs yielded similar results. The accuracy of each trial was modelled using GLMMs with a logit link function. LMMs are more flexible than traditional repeated measures ANOVAs, as they relax the strict statistical assumptions of ANOVAs and result in a more precise estimation of standard errors of regression coefficients (Boisgontier & Cheval, 2016).

In the perceptual matching task, we used sum contrasts to code the two fixed factors, matching (matching or mismatching pairs based on the label and face) and association (with self or stranger), and their interaction in the (G)LMMs. Similarly, for the facial emotion recognition task, we used sum contrasts to code the two fixed factors, association (with self or stranger) and emotion categories (Happiness, Surprise, Fear, Sadness, Disgust, Anger), and their interactions in the (G)LMMs. The ANOVA-like omnibus tests for main effects and interaction are reported for all predictors, and *p*-values are computed based on Type III Wald tests. Post-hoc pairwise tests were conducted using the lsmeans function from the emmeans 1.7.3 package with Holm-Bonferroni adjustments.

Due to the independence of the trials from the same participant and using the same facial model, we started with a crossed random effects structure for both participant and facial models, following the recommendation of Baayan et al. (2008). In order to assess the degree to which variance was explained by each random effect structure, ICC coefficients were calculated for the random effect structure by-participant and by-facial models in the null model (without any fixed factors). The ICC coefficients indicated that there was no substantial variation of both RTs and accuracy within identical facial models (< 0.1%), demonstrating that adding the random structure for facial models was not necessary (McNabb & Murayama, 2021). Therefore, we opted to include only the random effects structure by-participant. The random slopes of all predictors and random intercepts were determined using backward model selection according to the likelihood-ratio test (Matuschek et al., 2017). The model reduction procedure started with the full model with random intercept and random slopes for all fixed factors (Barr et al., 2013). We defined a set of reduced models by excluding one of the random slopes. One reduced model was selected when the result of the likelihood-ratio test was not significant compared to a more complex model. The model reduction procedure was repeated until a more complex model was selected or all the random slopes were excluded. Models that failed to converge were not considered in this procedure.

#### Additional analyses using Bayesian methods

A common critique of frequentist null-hypothesis significance testing (NHST) is that researchers often fail to obtain evidence that supports the null hypothesis (Dienes, 2014). As a consequence, evidence of no effect and data that is insufficient to detect an effect cannot be distinguished. One of the great benefits of Bayesian analysis is that it provides an estimate of how strongly the empirical results support either the null or the alternative hypothesis (Nathoo & Masson, 2016). Here, we performed additional Bayesian analyses to complement the results achieved by frequentist analysis.

Following the approach of Muth et al. (2018), we fitted Bayesian LMMs and Bayesian GLMMs separately for RTs and accuracy. We used the stan_lmer and stan_glmer functions in the rstanarm 2.21.3 package and specified the same random effect structure as the best model from the model selection procedure in the frequentist analysis. For the prior distributions, we used an unbiased weakly informative prior, which is equivalent to L2 regularization. To evaluate the strength of evidence for or against the entire fixed factor instead of each contrast coding, we used a model comparison approach to compare models including one fixed factor with models without that factor, similar to forward regression. For example, for self-association, we compared the model with this factor against the null model, and for the interaction between self-association and the matching factor, we compared the model with the interaction term against the model without the interaction. Therefore, we defined a set of models by including one of the fixed effects of factors in the null model. We sampled the joint posterior distribution for each model by running sixteen Monte Carlo Markov Chains (MCMCs) at 8000 iterations. The first half of the samples were discarded as warm-up samples. All models had $\hat{R}$ values lower than 1.1 (Gelman & Rubin, 1992), and all chains mixed and reached stationary distributions by visual inspection, indicating that the models converged well. Because the Bayesian analysis was intended to supplement the weakness of the NHST in null hypothesis testing, we calculated the Bayes Factors (${\text{BF}}_{01}$) as output, which indicates the ratio of the marginal likelihoods under the null hypothesis (excluding the factor, H0) and the alternative hypothesis (including the factor, H1) based on the data from this study. Based on Jeffreys's (1998) widely used evidence quality scale, a ${\text{BF}}_{01}$ > 3 indicates substantial evidence in favor of H0, and a ${\text{BF}}_{01}$ > 100 shows decisive evidence for H0.

## Results

### Perceptual matching task

After applying the backward model selection procedure with the likelihood-ratio test, we arrived at the following final LMM for RT in the perceptual matching task, which was specified as $\ln \,{\text{RTs }}\sim {\text{matching}} * {\text{association }} + (1 + {\text{matching}} + {\text{association}} | {\text{participant }})$. The ANOVA-like omnibus tests of the predictors revealed significant main effects of matching, $\chi^{2} \left( 1 \right)$ = 168.37, *p* < 0.001, and association, $\chi^{2} \left( 1 \right)$ = 126.80, *p* < 0.001, as well as a significant interaction between the two factors, $\chi^{2} \left( 1 \right)$ = 112.75, *p* < 0.001. Additional Bayesian model comparison revealed very small Bayes factors (${\text{BF}}_{01}$ < 0.01) for all fixed factors and their interaction, suggesting that the data decisively supported the existing effects of all factors and their interaction, which confirms the result from the frequentist LMM.

**Fig. 3:**
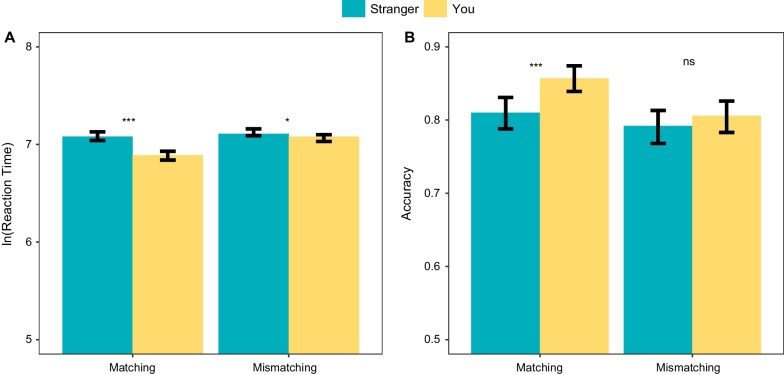
The results of the perceptual matching task. Error bars represent 95% confidence intervals

Follow-up simple-effect analyses (Fig. 3A) showed that RTs for the self-label were significantly quicker than those for the stranger-label, regardless of matching trails, *p* < 0.001, or mismatching trials, *p* = 0.032. However, the difference in RTs was larger for the matching trials (difference of $\ln {\text{RTs}}$ = 0.20) than for the mismatching trials (difference of $\ln {\text{RTs}}$ = 0.03).

Regarding accuracy in the perceptual matching task, the final GLMM after the selection was ${\text{ACC}} \sim {\text{matching}} * {\text{association}} + (1 + {\text{matching}} + {\text{association}} | {\text{participant }})$. The results showed that both fixed-effect factors and their interaction were significant, with matching, $\chi^{2} \left( 1 \right)$ = 21.02, *p* < 0.001, and association, $\chi^{2} \left( 1 \right)$ = 14.59, *p* < 0.001, having a significant effect and the interaction term, $\chi^{2} \left( 1 \right)$ = 8.76, *p* = 0.003, being significant as well. The complementary Bayesian GLMMs comparison revealed a similar pattern, with the Bayes factor associated with the factor matching decisively supporting the alternative hypothesis (${\text{BF}}_{01}$ < 0.01), indicating strong evidence of the difference between matching and mismatching trials. Although there was a significant difference in accuracy between the self-label and other-label trials, the Bayesian analysis provided weak evidence only (${\text{BF}}_{01}$ = 0.45), indicating that the data just slightly favored this difference over no difference. Regarding the interaction term, the evidence was weaker (${\text{BF}}_{01}$ = 0.72), although it suggested the existence of an interaction effect (Table 1).

**Table 1:** Estimates of perceptual matching task

Predictors	Ln (reaction time)				Accuracy			
	Estimates	CI	*p*	${\text{BF}}_{01}$	Odds ratios	CI	*p*	${\text{BF}}_{01}$
*Fixed effects*								
(Intercept)	7.04	7.00–7.08			4.51	4.11–4.95		
Matching	0.05	0.05–0.06	** < .001**	< .01	1.12	1.07–1.17	** < .001**	0.45
Association	0.06	0.05–0.07	** < .001**	< .01	0.90	0.85–0.95	** < .001**	< .01
Interaction	0.04	0.03–0.05	** < .001**	< .01	0.94	0.90–0.98	**.003**	0.72
*Random effects*								
*σ*^2^	0.20				3.29			
*τ*_00_	0.10 _Participant_				0.44 _Participant_			
*τ*_11_	0.01 _Participant: matching_				0.02 _Participant: matching_			
	0.01 _Participant: association_				0.07 _Participant: association_			
Marginal R2/conditional R2	0.025/0.368				0.007/0.146			

Post-hoc comparisons revealed higher accuracy (Fig. 3B) for the self-labeled compared to the stranger-labeled faces, but only within the matching trials, *p* < 0.001, and not within the mismatching trials, *p* = 0.183, which is consistent with previous studies.

#### Facial emotion recognition task with composite faces

**Fig. 4:**
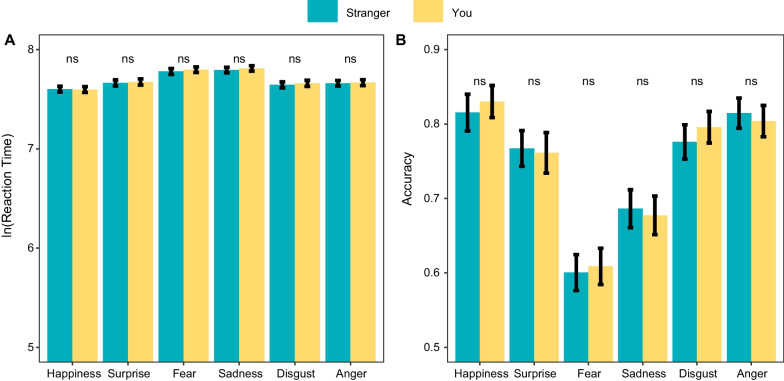
The results of the facial emotion recognition task. Error bars represent 95% confidence intervals

Regarding response times in the emotional expression recognition task, the model selection procedure identified the following final LMM $\ln {\text{RTs }}\sim {\text{association }}*{\text{emotion categories}} + (1 + {\text{association}} + {\text{emotion categories}} | {\text{participant}} )$. The only significant main effect was that of emotion categories, $\chi^{2} \left( 5 \right)$ = 814.76, *p* < 0.001. Confirming the results of the frequentist analysis, the Bayes factor was very small, ${\text{BF}}_{01}$ < 0.01, indicating strong evidence in favor of including the main effect of emotion. Post-hoc analysis (Fig. 4A) revealed that happy expressions were recognized most quickly ($M_{{{\text{RTs}}}}$ = 1895 ms), followed by disgust ($M_{{{\text{RTs}}}}$ = 1991 ms), anger ($M_{{{\text{RTs}}}}$ = 2022 ms), and surprise expressions ($M_{{{\text{RTs}}}}$ = 2031 ms), while fear ($M_{{{\text{RTs}}}}$ = 2323 ms) and sadness ($M_{{{\text{RTs}}}}$ = 2348 ms) were recognized more slowly than all other emotions. These results are consistent with many previous studies on facial emotion recognition (e.g., Mancini et al., 2018). However, the main effect of association ($\chi^{2} \left( 1 \right)$ = 2.92, *p* = 0.087) and its interaction with emotion categories ($\chi^{2} \left( 5 \right)$ = 1.29, *p* = 0.936) were all not significant. The Bayesian model comparison also provided strong evidence supporting the null hypothesis, with ${\text{BF}}_{01}$ > 100 for both the effects of self-association and its interaction with emotion categories.

Accuracy in this task was further tested using the GLMM. After model selection, the final retained model was ${\text{ACC }}\sim {\text{ association }}*{\text{emotion categories}} + (1 + {\text{ association}} + {\text{emotion categories}} |{\text{ participant}})$. Results again showed a significant main effect of emotion categories, $\chi^{2} \left( 5 \right)$ = 648.89, *p* < 0.001. Bayes factor model comparison decisively supported the alternative hypothesis, with ${\text{BF}}_{01}$ < 0.01, suggesting differences in accuracy between different emotion categories. A similar pattern to that seen in the response time results emerged in post-hoc analysis (Fig. 4B): happy expressions were recognized most accurately ($M_{{{\text{ACC}}}}$ = 0.86), followed by anger ($M_{{{\text{ACC}}}}$ = 0.84), disgust ($M_{{{\text{ACC}}}}$ = 0.81), and surprise expressions($M_{{{\text{ACC}}}}$ = 0.79), whereas sadness ($M_{{{\text{ACC}}}}$ = 0.70) and fear($M_{{{\text{ACC}}}}$ = 0.62) were recognized less accurately. Again, there was no significant main effect of association ($\chi^{2} \left( 1 \right)$ = 1.04, *p* = 0.307) or interaction between self-association and emotion categories ($\chi^{2} \left( 5 \right)$ = 8.40, *p* = 0.135). With the complementary Bayesian approach, we found strong evidence (${\text{BF}}_{01}$ > 100) in favor of the null hypothesis for both the main effect of the fixed factor association and its interaction with different emotion categories (Table 2).

**Table 2:** Estimates of facial emotion recognition task

*Predictors*	Ln (response time)				Accuracy			
	Estimates	CI	*p*	${\text{BF}}_{01}$	Odds ratios	CI	*p*	${\text{BF}}_{01}$
*Fixed effects*								
(Intercept)	7.65	7.62–7.67			3.50	3.23–3.80		
Association	− 0.01	− 0.01–0.00	.087	> 100	0.98	0.95–1.02	.307	> 100
Emotion categories			** < .001**	< .01			**.003**	< .01
Interaction			.936	> 100			.135	> 100
*Random effects*								
*σ*^2^	0.09				3.29			
*τ*_00_	0.04 _Participant_				0.40 _Participant_			
*τ*_11_	0.01 _Participant: association_				0.02 _Participant: association_			
	0.01 _Participant: emotion categories1_				0.15 _Participant: emotion categories1_			
	0.01 _Participant: emotion categories2_				0.17 _Participant: emotion categories2_			
	0.01 _Participant: emotion categories3_				0.11 _Participant: emotion categories3_			
	0.01 _Participant: emotion categories4_				0.30 _Participant: emotion categories4_			
	0.01 _Participant: emotion categories5_				0.15 _Participant: emotion categories5_			
Marginal R2/conditional R2	0.044/0.351				0.053/0.197			

The predictor association and interaction being studied has more than two levels, making it impossible to estimate a single value

## Discussion

Processing self-related information and emotional facial expressions are both essential to human social interaction. However, while numerous studies have focused on self-related information, few have explored how self-relevance influences facial emotion recognition. To eliminate the potential influence of confounding factors such as familiarity and overlearning in self-related research, we implemented a self-association paradigm to investigate how self-relevance influences the cognitive processing of facial expressions of emotion. Given previous methodological criticism regarding the familiarity effect of stimulus and the strong relationship between face identity and facial emotion processing, our first goal was to replicate the self-association paradigm to be used to extend self-relevance to facial information processing (Woźniak & Knoblich, 2019), using a large and diverse sample. Our second goal was to examine whether self-relevance would also modulate the processing of emotional facial expressions in a composite faces paradigm with six emotion categories.

### Self-relevance in the domain of face processing

As hypothesized, our results successfully replicated the effect of self-relevance on face processing reported in previous studies (Woźniak & Knoblich, 2019). In the perceptual matching task, both frequentist and Bayesian analyses consistently showed that participants reacted more accurately and quickly when an unknown facial stimulus was associated with their self-label in the matching trials, indicating that self-relevance facilitates face processing. Our study provides new, robust evidence for this phenomenon, given the large and diverse sample studied. Furthermore, we asked participants to evaluate multiple face models (four models per association condition) to test whether the self-relevance for face processing can be generalized across multiple stimuli. This goes beyond previous studies which often used only one face model per association condition (e.g., Payne et al., 2017).

Similar to previous studies, we did not observe a significant effect of self-relevance on accuracy in the mismatching trials. It is also in line with the literature that the effect size of self-relevance on reaction times was smaller in the mismatching trials compared to the matching trials. One explanation for these findings is that the unfamiliarity of the social label ("stranger" compared to "self") may have suppressed the effect of self-relevance (Woźniak & Knoblich, 2019). For example, studies have shown that the effect of self-relevance is weaker when using a foreign language social label compared to a native language label (Ivaz et al., 2016, 2019). However, as previous studies emphasized, the suppression effect of the social label familiarity does not negate the effect of self-relevance in matching trails, because the prioritization of self-associative processing can also be observed even without any social label (Lee et al., 2021; Woźniak & Knoblich, 2019). Here, we demonstrated a significant effect of self-association even when accounting for the variance explained by matching or mismatching trials. This indicates the robustness of evidence on self-prioritization in face processing.

### On the specificity of emotion categories

Regarding the facial emotion recognition task, our results replicate the specificity of recognizing emotions of different categories. This specificity has been observed in studies using different multimodal psychophysiological data, such as electromyogram activity (Künecke et al., 2014), brain blood flow (Fusar-Poli et al., 2009), and ERPs (Recio et al., 2014). Specifically, we found that happiness was perceived most accurately and quickly, whereas fear and sadness were perceived less accurately and more slowly than other facial emotion expressions. These findings are consistent with previous studies that have used the same task (Calder et al., 2000; Durand et al., 2007) or other tasks to measure emotion recognition from faces (Wilhelm et al., 2014). Taken together, our findings support the methodological recommendation of O'Sullivan and Ekman (2004) to use stimuli from a variety of different emotion categories, rather than focusing on only one or two, when measuring facial emotion recognition performance.

One significant limitation of our study is that we only included one measurement paradigm of facial emotion recognition. Although the measures in our study encompassed all emotion categories, which is an improvement compared to previous research, the use of only one task limits the generalizability of our findings. In experimental research focusing on individual differences, the performance of a specific task is usually decomposed into task- and construct-specific sources of variance (Schmiedek et al., 2009). It is assumed that a change in the task-specific source of variance could lead to a different conclusion regarding the psychological construct of interest. To rule out this possibility, methodologists recommend using multiple cognitive tasks to minimize the influence of task-specific sources of variance (Schmiedek et al., 2014). A previous multivariate study summarized sixteen different tasks to measure facial emotion recognition (Wilhelm et al., 2014). Thus, a crucial next step in this study would be to use multiple tasks to measure emotion recognition and its relationship to the self.

### Self-relevance on the recognition of facial expressions

Contrary to our hypothesis, we found no evidence that associating the self with an unfamiliar face altered the recognition of facial emotion expressions. Surprisingly, we observed the same non-significant results regardless of whether we used accuracy or response times as an indicator. Furthermore, the large Bayes factor supporting the null hypothesis indicates that the absence of self-prioritization in the domain of emotion recognition cannot be attributed to a lack of statistical power or insufficient sample size. We also found non-significance and a Bayes factor strongly supporting the null hypothesis in the test of the interaction term between self-association and emotion categories, indicating the same pattern across all emotions. Thus, following the successful association of the self with unfamiliar faces, participants did not perceive any emotional expression displayed by the faces associated with self-label more quickly or more accurately than those labeled as “stranger”.

Our results do not conceptually replicate previous findings regarding self-relevance in the processing of facial emotion expressions. Previous studies have shown that self-association can prioritize the processing of happy faces in perceptual matching tasks (Constable et al., 2021; McIvor et al., 2021), a phenomenon known as self-positivity bias (Herbert et al., 2013). One possible methodological explanation for our contradictory results is the different experimental designs used in our study and previous studies. As discussed in the introduction, previous studies may have inherent design flaws because integrating emotional expressions in perceptual matching tasks instead of using an additional special measure, which can lead to ambiguous interpretations (Siebold et al., 2015). Higher performance in the perceptual matching task with emotional expressions, like the previous studies, can be attributed either to preferential processing of emotional stimuli or to a stronger association between emotional faces and the self. While some researchers may argue that the same explanation can be applied to interpret our findings of facial processing (the first goal in our study), we argue that previous studies have ruled out this possibility by analyzing facial processing-related ERP in the perceptual matching task (Woźniak et al., 2018).

Another possible methodological explanation for the observed discrepancy may be the intrinsic characteristics of this study's facial emotion recognition task. Although the facial emotion recognition paradigm used in this study has very good psychometric properties in the context of other tasks as well (Hildebrandt et al., 2015; Wilhelm et al., 2014), the composite face requires participants to view two emotional expressions simultaneously, which leads to interference induced by the distracting emotional expression. Furthermore, the standard procedure which was designed to overcome potential ceiling effects in recognizing prototypical expressions (Wilhelm et al., 2014) restricted specific emotions to fixed top or bottom positions, arguably limiting our opportunity to fully explore interactive relationships between emotion placements. Therefore, it is worthwhile to replicate the present study using alternative facial emotion recognition paradigms which challenge different processing mechanisms, for example the Emotion Hexagon test (Wilhelm et al., 2014).

Secondly, as mentioned above, considerable evidence supports the self-positivity bias, suggesting that self-relevance enhances the recognition of positive facial expressions. In the task we used, the studied emotion categories were predominantly negative (e.g., sadness, fear, disgust, and anger) rather than positive (e.g., happiness) (An et al., 2017), which could potentially confound the results when using the composite face as a stimulus, given that happiness stimuli were combined with a negative expression in the upper part of the face. This is because the negative facial expressions could partially suppress the boosting effect of self-relevance on the positive facial expressions. However, the post hoc analysis allows us to at least partly rule out this possibility. If the composition of positive and negative facial expressions confounded the effect of self-relevance, an interaction effect would likely occur, as the composition of two negative facial expressions should yield worse performance than the positive–negative composition (a condition in which the self-positivity bias would at least partially occur). However, no significant interaction was observed, and the Bayes factor supported the null hypothesis. Statistically, this finding indicates that the effect of self-relevance on the recognition of different facial emotions remained consistent across conditions. Again, future research is needed to apply multiple tasks to further evaluate these effects.

### Two possible theoretical explanations

Beyond the potential methodological explanations discussed above, two possible theoretical explanations can be considered as well to account for the evidence provided in this study supporting a rather parallel processing of self-relevance and facial emotion recognition.

The first possible theoretical explanation revolves around the differentiation between processing self-associated facial information and facial expressions of emotion. According to the prominent Bruce and Young's model of facial information processing (see Calder & Young, 2005), general face processing involves several stages: structural encoding, the establishment of face recognition units, person identity nodes, and semantic information units (Burton et al., 1990). Notably, emotion expression recognition shares only the initial stage (facial structural coding) and then dissociates from general face perception according to the model (Calder & Young, 2005). This dissociation has been supported by evidence from brain injury patients (Bruyer et al., 1983; Tranel et al., 1988; Young et al., 1993), by functional brain imaging (Sergent et al., 1994), and more recent larger individual differences research with a multitasks approach (Hildebrandt et al., 2015). Therefore, although self-relevance is known to modulate cognitive processing at an early stage (Humphreys & Sui, 2016), emotional expression processing might not benefit from this due to its separate processing route.

To further elucidate this explanation, we draw upon previous evidence from ERP studies. While research has demonstrated that the effect of self-relevance can be detected in the very early stage in the non-facial domain (Sui et al., 2023), findings from the facial domain indicated that self-associated faces are differentiated from other-associated faces only after 200 to 300 ms (Żochowska et al., 2021). This relatively late self-other discrimination in facial processing can be attributed to the access of person identity nodes and semantic information units in face processing. In contrast, many studies have reported that the amplitude difference between emotional prototypes can be found before 200 ms or more (Luo et al., 2010; Recio et al., 2014), despite some conflicting evidence. Thus, while self-association of faces can accelerate general face processing in-person identity nodes and semantic information units, emotion expression processing remains unaffected, as the separate route for facial expression has already recognized the emotion expressions. In essence, our findings can be interpreted as follows: self-relevance influenced the person identity nodes and semantic information units of self-associated faces but did not impact the recognition of different emotion expressions displayed by these faces. This might be because the routes of invariant vs. expression-related facial information processing are only overlapping in the early stages. Consequently, this explanation accounts for both the lack of evidence supporting a difference in emotion expression recognition between self-associated and other-associated faces in our study and the boosting effect of self-relevance on face processing, as demonstrated by Woźniak and Hohwy (2020) and Woźniak and Knoblich (2019).

However, the present findings provide no direct evidence for this theoretical explanation, as we relied solely on response accuracy and response times as indicators and they cannot provide detailed information on the stages of cognitive processing (Heitz, 2014). This limitation has motivated researchers to employ different cognitive and psychophysiological techniques, such as eye-tracking (Siebold et al., 2015), and ERPs (Schreiter et al., 2019; Woźniak et al., 2018) and should be considered in future research to address the above theoretical view. Two high-feasibility modeling approaches to behavioral data might be beneficial in disentangling the underlying psychological processes during emotion expression recognition as well. These are the drift–diffusion model (Stafford et al., 2020), or mouse tracking (Scherbaum & Dshemuchadse, 2020). In the future, both techniques could be used to explore the different stages in emotional expression processing, allowing for more direct evidence to test the above theoretical explanation.

The second theoretical explanation pertains to the complex structure of the self. While the distinct route for facial emotion processing provides a reasonable justification for why self-relevance may not influence the recognition of expressions, it remains difficult to reconcile this with the abundant evidence of a close relationship between the self and emotional facial expression processing. For instance, a body of literature suggests that participants recognize facial expressions better when their own faces are used as stimuli (Li & Tottenham, 2013). Although the familiarity and overlearning of stimuli may explain this finding, an alternative reason could be the different structure of the self. According to the consensus of self-related research, the self, as a complex structure, has different conceptualizations, including the “bodily” self and the “conceptual” self (Farmer & Tsakiris, 2012).

While self-relevance is a powerful tool for exploring processing biases toward self-related information, it is primarily applied to changes in the level of the “conceptual” self (Maister & Farmer, 2016). Neuroimaging studies have shown that the self-association paradigm recruits the ventromedial prefrontal cortex (vmPFC), which is more closely related to the conceptual self-related neural network (Humphreys & Sui, 2016) rather than the bodily self-related neural network (Tsakiris, 2010). In line with previous research indicating that self-association with an unknown face can alter facial representation at the conceptual level (Woźniak et al., 2018) but not at the bodily level (Payne et al., 2017), our study found facial processing was enhanced by self-association. However, unlike general face perception, the simulation theory of facial expression recognition suggests that successful emotion recognition from faces requires the activation of the sensorimotor cortex (Wood et al., 2016), which is a part of the bodily self-related neural network (Tsakiris, 2010) and more closely associated with the bodily self (Farmer & Tsakiris, 2012). This is supported by research demonstrating improved facial expression recognition performance through bodily self-manipulation using the enfacement paradigm (Maister et al., 2013). Therefore, it is understandable that we did not find evidence supporting the role of self-relevance in emotion recognition from faces because only the conceptual self was manipulated. Similar to the first explanation, the discrepancy between our findings and previous studies could also be reconciled within the same theoretical explanation.

However, like the first explanation, this explanation is not without its limitations. Some researchers may argue that the conceptual self and bodily self can co-influence each other. Previous research supports this argument, showing that manipulating the bodily self can affect the conceptual self and vice versa (Farmer & Tsakiris, 2012; Porciello et al., 2018). Therefore, it is possible that even a change in the conceptual self, such as in our study, could lead to similar effects as a change in the bodily self. However, our study's results suggest that this bidirectional relationship does not always hold. The conditions under which bidirectional relationships occur, and when they do not, remain unclear. It is possible that the relationship between the conceptual self and the bodily self is context-dependent, and that certain factors, such as the type of task or emotional stimuli used, may influence the direction and strength of this relationship (Porciello et al., 2018). Therefore, a future direction would be to use both self-association and enfacement paradigms to manipulate both the conceptual and bodily self and re-examine their influence on facial emotion recognition. This could help clarify the conditions under which bidirectional relationships occur and whether they are consistent across different contexts. Additionally, it could provide a more comprehensive understanding of the relationship between the conceptual and bodily self and their respective roles in emotion processing.

## Conclusion

Our study contributes to the understanding of how self-relevance influences the cognitive processing of facial expressions of emotion. In a large and diverse sample, by means of the self-association paradigm, we replicated the effect of self-relevance on face processing but did not find evidence to support that self-relevance influences facial emotion recognition performance. Two possible theoretical explanations were proposed to account for the lack of evidence, but further research with extended experimental designs and more comprehensive measures is necessary to fully understand these. Overall, our study adds to the literature on self and facial emotion processing, highlighting the need for further research to better understand the complex interplay between these two.

## Data Availability

The datasets generated and the code for the data analysis can be found on the Open Science Framework (OSF) website for this project at https://osf.io/4n6j7/

## Abbreviations

ERPs: Event-related potentials
2AFC: Two-alternative forced choice
RTs: Response times
LMMs: Linear mixed models
GLMMs: Generalized linear mixed models
ANOVA: Analysis of variance
NHST: Null-hypothesis significance testing
BF: Bayes factor
MCMCs: Monte Carlo Markov Chains
vmPFC: Ventromedial prefrontal cortex
